# Systematic review of the introduction, early phase study and evaluation of pyrocarbon proximal interphalangeal joint arthroplasty

**DOI:** 10.1371/journal.pone.0257497

**Published:** 2021-10-19

**Authors:** Paul Welford, Natalie S. Blencowe, Emily Pardington, Conor S. Jones, Jane M. Blazeby, Barry G. Main

**Affiliations:** 1 North Bristol NHS Trust, Bristol, United Kingdom; 2 Bristol Centre for Surgical Research, Department of Population Health Sciences, Bristol Medical School, Bristol, United Kingdom; 3 National Institute for Health Research Biomedical Research Centre at Bristol and Weston NHS Foundation Trust, Bristol, United Kingdom; 4 University Hospitals Bristol and Weston NHS Foundation Trust, Bristol, United Kingdom; 5 Bristol Medical School, University of Bristol, Bristol, United Kingdom; Medical University of Graz, AUSTRIA

## Abstract

**Background:**

In 2002 a pyrocarbon interphalangeal joint implant was granted Food and Drug Administration approval with limited evidence of effectiveness. It is important to understand device use and outcomes since this implant entered clinical practice in order to establish incremental evidence, appropriate study design and reporting. This systematic review summarised and appraised studies reporting pyrocarbon proximal interphalangeal joint arthroplasty.

**Methods:**

Systematic review of MEDLINE, EMBASE, SCOPUS, Web of Science, BIOSIS, CINAHL and CENTRAL from inception to November 2020. All study designs reporting pyrocarbon proximal interphalangeal joint arthroplasty in humans were included. Data extracted included information about study characteristics, patient selection, regulatory (gaining research ethics approval) and governance issues (reporting of conflicting interests), operator and centre experience, technique description and outcome reporting. Descriptive and narrative summaries were reported.

**Results:**

From 4316 abstracts, 210 full-text articles were screened. A total of 38 studies and 1434 (1–184) patients were included. These consisted of three case reports, 24 case series, 10 retrospective cohort studies and one randomised trial. Inclusion and exclusion criteria were stated in 25 (66%) studies. Most studies (n = 27, 71%) gained research ethics approval to be conducted. Six studies reported conflicting interests. Experience of operating surgeons was reported in nine (24%) and caseload volume in five studies. There was no consensus about the optimal surgical approach. Technical aspects of implant placement were reported frequently (n = 32) but the detail provided varied widely. Studies reported multiple, heterogenous outcomes. The most commonly reported outcome was range of motion (n = 37).

**Conclusions:**

This systematic review identified inconsistencies in how studies describing the early use and update of an innovative procedure were reported. Incremental evidence was lacking, risking the implant being adopted without robust evaluation. This review adds to evidence highlighting the need for more rigorous evaluation of how implantable medical devices are used in practice following licencing.

## Introduction

Arthritis of the hand affects approximately 15% of the adult population. The proximal interphalangeal joints (PIPJs) are commonly involved [1]. Whether caused by degenerative, inflammatory or post-traumatic arthritis, PIPJ destruction brings significant functional impairment [2]. Should unremitting pain and poor function persist despite non-operative treatments, patients may be offered surgery. Joint fusion is considered by some to represent the gold standard surgical treatment, but can cause a debilitating loss of hand function [3,4]. Such undesirable outcomes have nurtured an enduring interest in PIPJ arthroplasty [5].

Arthroplasty of the PIPJ was first reported in 1940 [6]. In early studies, major complications including skin breakdown, infection, periarticular fibrosis and bone resorption were reported [7,8]. Silastic implants, which promised biological inertness and theoretical increased durability, were subsequently introduced [9,10]. Pain relief was good but implant fracture, delayed infection and silicone lymphadenopathy were noted [11–14]. Pyrocarbon–an inert biomaterial with physical properties between those of graphite and diamond–has been used in heart valves since 1977 [15]. It was hypothesised to overcome these shortcomings. Theoretical advantages include high strength, low-friction and elasticity to allow for implant-bone stress transfer [15,16]. Pyrocarbon PIPJ arthroplasty (pPIPJa) was first performed in France in 1994 [17].

In 2002, the FDA authorised a pyrocarbon PIPJ implant (Ascension Orthopaedics, Texas, USA) under the ‘Humanitarian Device Exemption’, which permitted marketing and use without evidence of effectiveness [18]. Since that time, outcomes have been controversial but the implants remain in use [19,20].

In view of this history, an in-depth analysis of the development and evaluation of pPIPJa is valuable. It is hypothesised that this will help to understand and make available a summary of the current evidence for this device. It will provide information about how surgeons have used the device in practice, what governance procedures have been followed, how the intervention has evolved technically and how patient risk is managed throughout. This systematic review therefore aims to summarise and appraise the reporting of studies of pPIPJa from first-in-human to routine clinical practice.

## Methods

This systematic review identified published studies of pPIPJa and was conducted in line with the Preferred Reporting Items for Systematic reviews and Meta-analysis (PRISMA) [21]. Methods were based on a previously published protocol and are summarised below [22].

### Search strategy and study selection

A comprehensive search strategy (S1 File) was developed in collaboration with an information specialist. Our search strategy incorporated medical subject headings and keywords. Terms were included for ‘interphalangeal joint’, ‘arthroplasty’ and ‘prosthesis’. The MEDLINE and EMBASE (via OVID SP), SCOPUS, Web of Science, BIOSIS, CINAHL and the Cochrane Central Register of Controlled Trials were searched electronically from inception to 1^st^ November 2020. Reference lists from included studies were searched manually to identify additional relevant studies.

### Study eligibility

Searches were limited to studies involving humans. Preclinical studies were therefore excluded. All primary clinical research study designs (e.g. case reports, case series, and comparative studies) were included. Systematic reviews were not included in the final analysis, but their reference lists were cross-checked for additional eligible studies. Presentations and conference abstracts were excluded because of the high probability of incomplete data. Papers not written in English were excluded.

### Identification and selection of papers

Search results were uploaded to Endnote© reference management software and de-duplicated. Titles and abstracts were screened by at least two independent reviewers (PW, EP, CJ). The full text articles retained after screening were further assessed for eligibility by the same two authors. Any disputes regarding study inclusion were discussed and resolved with the senior author (BM). Reference lists of included papers were hand searched for additional relevant papers. Data from full text papers were extracted independently by at least two researchers (PW, EP, CJ).

### Data collection

Data were extracted using a customised data extraction form and included information about study characteristics, patient selection, regulatory and governance issues (e.g. informed consent), operator and centre experience, technique description and outcomes [22].

### Study characteristics

For each included study the year and journal of publication, study design, country of origin, number of participating centres, number, sex and age of patients, length of follow up, hypothesis and rationale were recorded.

### Patient selection

Study-specific inclusion/exclusion criteria and whether or not patients were consecutively recruited was recorded. This gives some indication of how surgeons use innovative devices in patients with more advanced disease or challenging morphology. Information about those patients eligible for pPIPJa, but who did not consent to the intervention was collected.

### Regulatory and governance arrangements

Information about governance approvals (for example, ethics committees or institutional review boards) and FDA implant authorisation status was recorded. This gives an indication of how surgeons viewed use of the recently approved device and its accompanying evidence. Where research protocols are prepared and employed it is usually associated with a higher degree of risk management and more transparent patient consent to receive an innovative procedure. Papers were analysed for whether the study protocol was modified after ethical approval and whether patients were specifically informed about the innovative nature of the procedure (and any subsequent modifications). Reports of any funding received from the manufacturer, or other potential conflicts of interest, were also documented.

### Operator and centre expertise

Caseload volume, type of centre (e.g. secondary or tertiary centre), and the number of surgeons undertaking pPIPJa were recorded. Reports of surgeon and team expertise with the procedure, including any descriptions of the learning curve, were extracted.

### Descriptions of interventions and co-interventions

Details of the technical steps reported in performing pPIPJa were recorded by developing a typology based on published work, in collaboration with a hand surgeon [23]. Descriptions of each step (such as incision, access, medullary canal opening and component broaching, trial implant position assessment via X-ray and range of motion, management of the central slip, and closure) were tabulated chronologically to identify whether there was clear reporting of what had been performed and whether modifications to the surgical technique had occurred over time. If modifications to the device or operative procedure were reported, these were noted alongside any reported rationale for that change in practice.

The reporting of co-interventions, including pre- and perioperative imaging; prescription of antibiotics; anaesthesia; analgesia; thromboprophylaxis; the use of perioperative fluids, lines, tubes or monitoring and post-operative rehabilitation was documented.

### Outcome selection, measurement and reporting

All outcomes reported in the included papers were extracted and categorised into the following groups: clinical (a clinician’s assessment of symptoms or signs); radiological outcomes (an assessor’s interpretation of X-rays); patient-reported (a report of the patient’s health condition that comes directly from the patient, without interpretation by a clinician or anyone else); process (the specific steps that lead to a particular outcome); cost and other economic outcomes; adverse events (an untoward medical occurrence as a result of the intervention); and implant removal. Whether or not authors designated primary and secondary outcomes was recorded. It was determined whether reported outcomes had been independently assessed, and defined. For patient reported outcomes, the instrument used was recorded along with whether or not it was validated, whether there was a reference to validation of the instrument and where in the study it had been reported. The total number of outcomes per study and the frequency of reporting for each outcome was recorded. The time scale for reporting adverse events, rationale for implant removal and the techniques used for removal were documented.

### Data synthesis and statistical analysis

Findings were summarised in a narrative synthesis with descriptive statistics presented where appropriate. The aim of the paper was to summarise and appraise the standards of reporting of studies of pyrocarbon proximal interphalangeal joint arthroplasty, from first published description to present day. As this study focussed on how an innovative surgical intervention was reported in the scientific literature and did not aim to make conclusions about the relative effectiveness of this intervention, compared to other treatments, meta-analyses were not performed. This methodology was based upon a published protocol and has been used to appraise the reporting of other innovative surgical procedures

## Results

### Study characteristics

**Fig 1:**
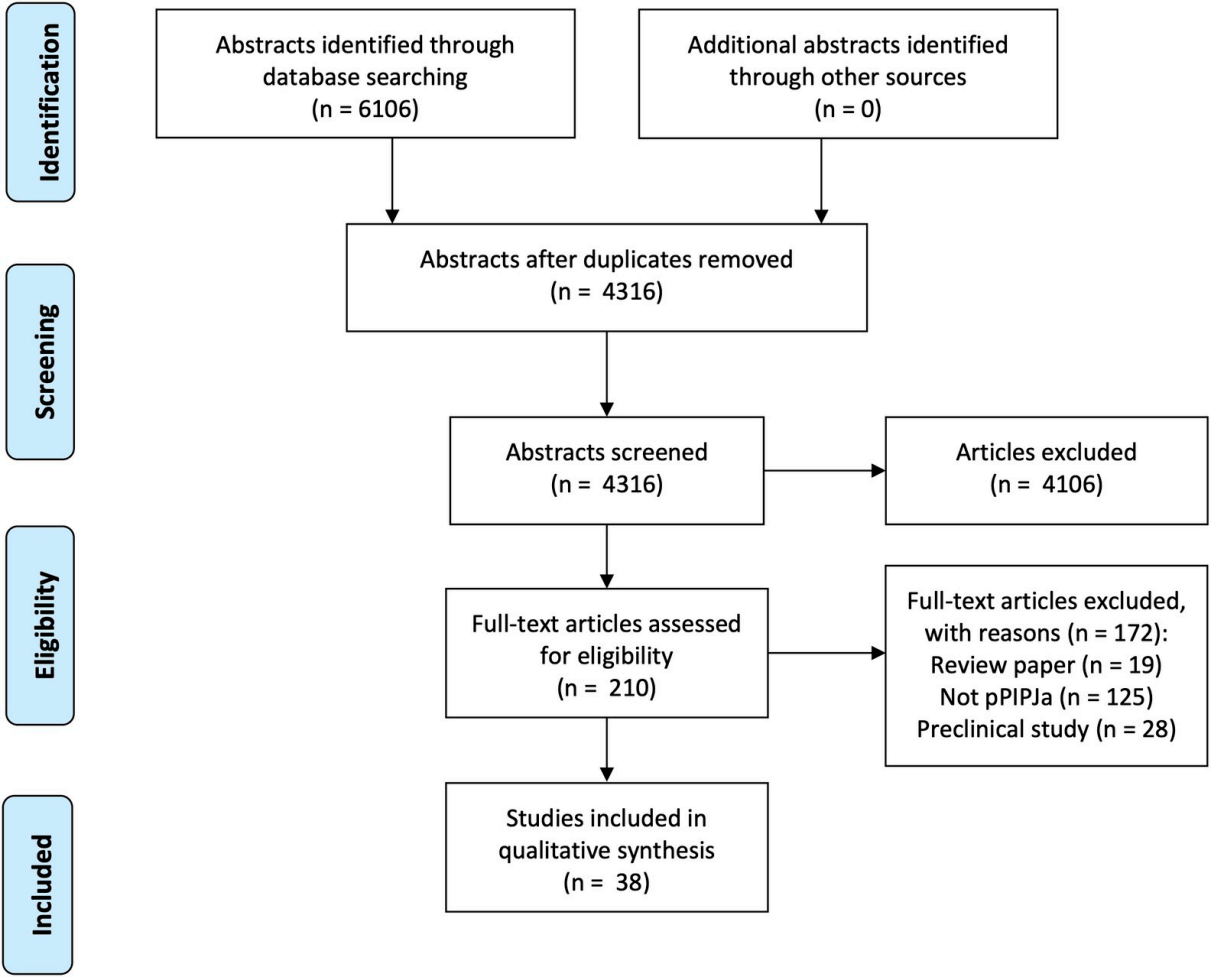
Preferred Reporting of Systematic Reviews and Meta-Analyses (PRISMA) flowchart.

Searches identified 6106 abstracts and, after removing duplicates, 4316 were screened (Fig 1). Excluded abstracts did not correspond to studies of pPIPJa. For example, many studies of Poly Implant Protheses (PIP) breast implants were excluded during abstract screening. Similarly, during full-text screening, the most frequent reason for exclusion was that a paper did not report a study of pPIPJa (n = 125). A total of 38 studies, published between 2006 and 2020, were included in the final analysis [3,18–20,24–57]. These comprised three case reports, 24 case series, 10 retrospective cohort studies and one randomised controlled trial (RCT) (Table 1). There were 22 studies from North America, 15 from Europe/UK, and one from South Africa. Three of the 38 studies were multicentre. A clear *a priori* rationale for the study was reported in 33 papers. The most commonly stated research rationale was to test for safety, efficacy or adverse events (n = 28). Five studies did not state any rationale for the research. A total of 1434 patients were included (range = 1–184), of whom 68% were female. The mean age of patients was 57 years (16–104). Length of follow up was reported by 36 studies, with a range of between six weeks and 130 months.

**Table 1:** Characteristics of included studies.

Author (reference)	Year	Country	Type of study	Single or multi centre	Patients (N)	Average follow up period
Herren et al [24]	2006	Switzerland	Non-comparative study	single	14	19 months
Nunley et al [25]	2006	USA	Non-comparative study	single	5	17 months
Tuttle and Stern [26]	2006	USA	Non-comparative study	single	8	13 months
Bravo et al [27]	2007	USA	Non-comparative study	single	35	37 months
Skie et al [28]	2007	USA	Case report	single	1	6 weeks
Branam et al [29]	2007	USA	Retrospective cohort study	single	25	31 months
Meier et al [30]	2007	Germany	Non-comparative study	single	20	15 months
Chung et al [18]	2009	USA	Non-comparative study	single	14	12 months
Wijk et al [31]	2010	Sweden	Non-comparative study	single	43	23 months
Sweets and Stern [20]	2011	USA	Non-comparative study	single	17	55 months
Henry [32]	2011	USA	Case report	single	2	33 months
Pritsch and Rizzo [19]	2011	USA	Retrospective cohort study	single	59	27 months
Daecke et al [33]	2012	Germany	Randomised trial	multi	43	35 months
Ono et al [34]	2012	USA	Non-comparative study	Single	13	44 months
Watts et al [35]	2012	UK	Non-comparative study	single	72	60 months
McGuire et al [36]	2012	South Africa	Non-comparative study	single	45	27 months
Hutt et al [37]	2012	UK	Non-comparative study	single	15	74 months
Mashhadi et al [38]	2012	UK	Non-comparative study	single	16	48 months
Heers et al [39]	2013	Germany	Non-comparative study	single	10	99 months
Desai et al [40]	2014	UK	Non-comparative study	single	14	42 months
Van Nuffel et al [41]	2014	Belgium	Retrospective cohort study	single	32	Not reported
Reissner et al [42]	2014	Switzerland	Non-comparative study	single	14	9.7 months
Tagil et al [43]	2014	Sweden	Non-comparative study	single	65	Not reported
Vitale et al [44]	2015	USA	Retrospective cohort study	single	79	67 months
Storey et al [45]	2015	UK	Non-comparative study	single	36	85 months
Dickson et al [3]	2015	UK	Non comparative study	single	72	118 months
Pettersson et al [46]	2015	USA	Non comparative study	multi	38	55 months
Wagner et al [47]	2015	USA	Retrospective cohort study	single	49	63 months
Wagner et al [48]	2015	USA	Retrospective cohort study	single	16	63 months
Rinkinen et al [49]	2016	USA	Case report	single	1	9 months
Srnec et al [50]	2018	USA	Retrospective cohort study	single	136	67 months
Wagner et al [51]	2018	USA	Non-comparative study	single	109	62 months
Duncan et al [52]	2018	USA	Non-comparative study	single	20	33 months
Wagner et al [53]	2019	USA	Retrospective cohort study	single	184	77 months
Tranchida et al [54]	2019	USA	Retrospective cohort study	multi	66	4 months
Selig et al [55]	2020	Germany	Non-comparative study	single	27	116 months
Wagner et al [56]	2020	USA	Retrospective cohort study	single	NR	71 months
Mora et al [57]	2020	USA	Non-comparative study	single	19	77 months

### Patient selection

A priori inclusion and exclusion criteria were stated in 25 (66%) studies. Indications for surgery included osteoarthritis in 924/1434 (65%) patients, post-traumatic arthritis in 320/1434 (22%) patients, and inflammatory arthritis in 190/1434 (13%) patients. Twelve (32%) studies explicitly reported that consecutive patients were included. No studies reported how many patients declined pPIPJa or stated what happened to these patients, nor reported what happened to patients not meeting the inclusion criteria.

### Regulatory and governance arrangements

Institutional review board or ethics committee approval was reported in 27 (71%) studies. Eleven (29%) described individual patient consent. Five (13%) studies included a statement on FDA authorisation status of the implant. No study reported that patients were notified of the innovative nature of the procedure, nor were there any reports of patients being provided with additional information, such as written material about the implant. Although six studies reported modification of the surgical procedure after the study commenced, no study reported that patients had been notified of these modifications or that ethical approvals were amended. Conflict of interest statements were published in 33 (87%) studies. Six studies reported potential conflicts of interests: authors were either paid consultants, design surgeons or in receipt of royalties from the implant manufacturer [20,27,31,43,44,47]. Funding statements were reported in 27 (71%) studies. One study was partly supported by the implant manufacturer (Table 2) [27].

**Table 2:** Overview: Patient selection, regulatory and governance arrangements, operator expertise and recommendations.

First author (reference)	Year	Conflict of interest statement	Funding statement	Ethics approval statement	Patient consent statement	Inclusion and exclusion criteria reported	Consecutive recruitment of patients	Surgical experience criteria statement	Modifications reported after study commenced	Recommendation for future use of pPIPJa
Herren [24]	2006	X	X	X	X	✓	X	✓ (generic)	Yes	Ongoing innovation
Nunley [25]	2006	✓	✓	✓	✓	✓	✓	✓ (generic)	No	Discontinuation
Tuttle [26]	2006	✓	✓	✓	X	X	X	X	No	Further evaluation
Bravo [27]	2007	✓!	✓!	✓	X	✓	X	X	No	Further evaluation
Skie [28]	2007	X	X	X	X	X	X	X	No	Continued use
Branam [29]	2007	✓	✓	✓	✓	✓	X	X	No	No recommendation
Meier [30]	2007	X	X	X	X	✓	X	X	No	No recommendation
Chung [18]	2009	✓	✓	✓	✓	✓	✓	X	No	Further evaluation
Wijk [31]	2010	✓!	X	✓	X	X	X	X	No	Continued use
Sweets [20]	2011	✓!	✓	✓	X	✓	X	X	Yes	Discontinuation
Henry [32]	2011	✓	X	X	X	✓	X	X	No	Further evaluation
Pritsch [19]	2011	✓	✓	✓	X	✓	X	X	No	Further evaluation
Daecke [33]	2012	✓	✓	✓	✓	✓	X	✓ (specific)	No	No recommendation
Ono [34]	2012	✓	✓	✓	✓	✓	✓	X	No	Further evaluation
Watts [35]	2012	✓	✓	✓	X	X	X	X	No	Continued use
McGuire [36]	2012	✓	✓	✓	X	X	✓	X	Yes	Routine practice
Hutt [37]	2012	✓	✓	X	X	✓	X	X	Yes	Further evaluation
Mashhadi [38]	2012	✓	✓	X	X	X	X	X	No	Routine practice
Heers [39]	2013	✓	✓	X	X	✓	X	X	No	Discontinuation
Desai [40]	2014	X	X	X	X	✓	✓	X	No	Routine practice
Van Nuffel [41]	2014	X	✓	X	X	✓	X	X	No	Discontinuation
Reissner [42]	2014	✓	✓	✓	✓	✓	X	X	Yes	Discontinuation
Tagil [43]	2014	✓!	✓	X	X	X	✓	X	Yes	Further evaluation
Vitale [44]	2015	✓!	X	✓	✓	✓	X	X	No	Discontinuation
Storey [45]	2015	✓	✓	X	X	X	X	✓ (specific)	No	Routine practice
Dickson [3]	2015	✓	✓	✓	X	X	✓	X	No	No recommendation
Pettersson [46]	2015	✓	✓	✓	X	✓	X	X	No	Routine practice
Wagner [47]	2015	✓!	X	✓	X	✓	✓	X	No	Ongoing innovation
Wagner [48]	2015	✓	✓	✓	X	✓	✓	X	No	No recommendation
Rinkinen [49]	2016	✓	✓	✓	✓	X	X	X	No	No recommendation
Srnec [50]	2018	✓	✓	✓	X	✓	✓	✓ (generic)	No	Routine practice
Wagner [51]	2018	✓	X	✓	X	✓	X	X	No	Continued use
Duncan [52]	2018	✓	X	✓	✓	X	X	✓ (generic)	No	Routine practice
Wagner [53]	2019	✓!	X	✓	X	✓	X	X	No	Further evaluation
Tranchida [54]	2019	✓	✓	✓	✓	✓	X	X	No	Further evaluation
Selig [55]	2020	✓	✓	✓	✓	X	✓	✓ (generic)	No	Routine practice
Wagner [56]	2020	✓	✓	✓	X	✓	✓	✓ (generic)	No	Continued use
Mora [57]	2020	✓	✓	✓	X	X	X	✓ (generic)	No	Further evaluation

Key

✓: Included.

X: Not included.

!: Possible conflict of interest reported.

### Operator and centre expertise

Caseload volume was reported in five studies (range = 4–25 patients per centre per year). No studies reported the type of centre (e.g. secondary or tertiary). The number of surgeons performing the procedure was reported in 27 papers (range = 1–11). Only two studies included pre-specified eligibility criteria for surgeons to undertake the procedure, while a further seven made a generic statement about the expertise of participating surgeons (Tables 2 and 3). No paper reported information about learning curves, or the expertise or training of the wider surgical team (e.g. nurses and anaesthetists).

**Table 3:** Statements of expertise and training of participating surgeons.

Author (reference)	Statement
Herren et al [24]	“Surgery was carried out entirely by two experienced surgeons.”
Nunley et al [25]	“Both senior authors (M.I.B. and C.A.G.) together performed all surgeries. . .”
Daecke et al [33]	“Five senior hand surgeons performed the operations. All of them were trained on cadaveric specimens before the study began to establish a standardized procedure for the implantations.”
Storey et al [45]	“The level of expertise of the surgeon who performed all the cases is level III according to the expertise classification of Tang.”
Srnec et al [50]	“The average surgeon expertise was level 4.8 in both the single and multi-digit groups. Furthermore, two surgeons of expertise level 5 were responsible for 70% of single digit and 75% of multi-digit PIP joint arthroplasty.”
Duncan et al [52]	(operations)”were performed by 3 fellowship-trained hand surgeons at our institution.”
Mora et al [57]	(surgery was performed by) “Certificate of Added Qualifications-certified orthopedic hand surgeons…”
Selig et al, 2020 [55]	“Various senior surgeons of one hand surgical department performed the surgeries.”
Wagner et al, 2020 [56]	“All of the surgeons operated on both border and middle digits and level of expertise of these surgeons are of or above 3 (Tang and Giddins, 2016).”

### Technique description

The level of detail provided for steps in pPIPJa varied greatly (Table 4). Six studies included no details of the surgical procedure, although one cited a previous study describing the technique [31,34,43,50]. A technique guide supplied by the implant manufacturer was cited by three studies [25,26,38]. In terms of specific components of the procedure, surgical approach was described in 32 (84%) studies. The dorsal approach was most common (n = 24), while two studies reported an exclusively volar approach. A variety of approaches, including lateral were reported in six studies but only two reporting a rationale for this. In one study, “no instrumentation (was) available for volar approach at the beginning of the series” [24] whereas another study aimed to compare outcomes between the dorsal and volar approach [54]. One study reported that surgical approach was “decided by the treating surgeon” [3]. Method of surgical access was described in 29 (76%) studies. The most commonly reported access method was extensor-tendon splitting (n = 10), followed by Chamay (a V-shaped incision that allows the central slip to be reflected distally; n = 8) [58].

**Table 4:** Reporting of the key components of pyrocarbon PIPJ arthroplasty in included papers.

First author (reference)	Incision location	Access type	Medullary canal opening & alignment	Proximal osteotomy & component broaching	Trial implant position assessed via X-rays & ROM	Middle phalanx exposure, distal surface preparation, distal component broaching	Management of central slip	Trial implant position assessed via X-rays, ROM and lateral joint laxity	Definitive implant size evaluated	Central slip formally reattached	Closure
Herren [24]	✓	✓	X	X	X	X	X	X	X	X	X
Nunley [25]	✓	✓	✓	✓	✓	✓	✓	✓	✓	✓	✓
Tuttle [26]	✓	✓	✓	✓	✓	✓	✓	✓	✓	X	✓
Bravo [27]	✓	✓	✓	✓	✓	✓	✓	✓	✓	✓	✓
Skie [28]	✓	✓	✓	✓	✓	X	✓	✓	X	✓	X
Branam [29]	✓	✓	✓	✓	✓	✓	✓	✓	✓	X	✓
Meier [30]	✓	✓	✓	✓	✓	✓	✓	✓	✓	✓	✓
Chung [18]	✓	✓	✓	✓	✓	✓	✓	X	✓	X	✓
Wijk [31]	✓	✓	✓	✓	✓	✓	✓	✓	✓	✓	✓
Sweets [20]	✓	✓	X	X	X	X	✓	X	X	X	X
Henry [32]	✓	✓	✓	✓	X	X	✓	X	✓	X	X
Pritsch [19]	✓	✓	X	X	X	X	X	X	X	X	X
Daecke [33]	✓	✓	✓	✓	✓	✓	✓	✓	✓	✓	✓
Ono [34]	X	X	X	X	X	X	X	X	X	X	X
Watts [35]	✓	✓	X	X	X	X	X	X	X	X	X
McGuire [36]	✓	✓	X	✓	✓	✓	✓	✓	✓	✓	✓
Hutt [37]	✓	✓	✓	✓	X	X	✓	X	X	X	X
Mashhadi [38]	✓	✓	X	X	X	X	✓	X	X	X	X
Heers [39]	✓	✓	X	X	X	X	X	X	✓	X	X
Desai [40]	✓	✓	✓	✓	✓	✓	✓	✓	✓	✓	✓
Van Nuffel [41]	✓	✓	✓	✓	✓	X	✓	X	X	✓	✓
Reissner [42]	✓	✓	X	X	X	X	✓	X	X	X	X
Tagil [43]	X	X	X	X	X	X	X	X	X	X	X
Vitale [44]	✓	✓	X	X	X	X	X	X	X	X	X
Storey [45]	✓	✓	✓	X	X	X	✓	X	✓	✓	✓
Dickson [3]	✓	✓	X	X	X	X	X	X	X	X	X
Pettersson [46]	✓	✓	✓	✓	✓	✓	✓	✓	✓	✓	✓
Wagner [47]	✓	X	X	X	X	X	X	X	X	X	X
Wagner [48]	X	X	✓	X	X	X	X	X	X	X	X
Rinkinen [49]	✓	✓	✓	N/A	N/A	N/A	✓	N/A	N/A	X	X
Srnec [50]	X	X	X	X	X	X	X	X	X	X	X
Wagner [51]	✓	✓	X	X	X	X	X	X	X	X	X
Duncan [52]	✓	✓	✓	✓	✓	✓	N/A	✓	✓	N/A	✓
Wagner [53]	X	X	X	X	X	X	X	X	X	X	X
Tranchida [54]	✓	X	X	X	X	X	X	X	X	X	X
Selig [55]	✓	✓	X	X	X	X	✓	X	X	X	X
Wagner [56]	✓	X	X	X	X	X	X	X	X	X	X
Mora [57]	X	X	X	X	X	X	X	X	X	X	X

Key

✓: Included.

X: Not included.

N/A: Not applicable.

### Co-interventions and technique modifications

With the exception of post-operative hand therapy, descriptions of co-interventions were frequently absent. Pre-operative imaging was reported in seven papers, and pre-operative antibiotics in two. Anaesthetic techniques were reported in five articles and peri-operative imaging in six. No studies reported analgesia, thromboprophylaxis, or the use of any other medications. The use of perioperative fluids, lines, tubes or monitoring was not reported. Post-operative hand therapy was reported in 27 studies. There was a wide variation in both the provision of post-operative rehabilitation and in the level of detail reported. Bravo et al (2007), for example, published a detailed description of their rehabilitation protocol with a summary table [27]. By comparison, Van Nuffel et al (2014) included a short paragraph that concluded, “routinely, no physiotherapy was given” [41].

Five studies specified at the outset that the authors had investigated a modification, refinement or adaptation of pPIPJa. Modifications included a volar approach, and using the pyrocarbon implant both for revision surgery and hemiarthroplasty. Six studies reported modifications to pPIPJa technique after the study had started (Table 2). Of these, four changed their surgical approach [20,24,36,42] and another switched from total joint arthroplasty to replacement of the distal joint surface only [43]. Only one study reported a rationale for procedural modifications that occurred after the study commenced [37]. That study introduced intraoperative fluoroscopy and started to splint patients’ digits in slight flexion postoperatively, aiming to “improve positioning and prevent problems with hyperextension” postoperatively.

### Outcome selection, measurement and reporting

A total of 46 different outcomes (Table 5) were reported across the included studies (range = 2–25). Twelve outcomes were stated in the methods section only, but not reported elsewhere in the paper. Watts et al (2012), for example, stated in their methods that grip strength was evaluated, but this outcome was not reported in the results, discussion or figures [35]. Only two studies defined the primary outcome of interest at the outset [37,54].

**Table 5:** Outcome reporting in included studies.

Category of outcome		No. studies reporting outcome n = 38 (%)
**Clinical**	**Any clinical outcome**	**37 (97)**
	Range of motion	37 (97)
	Grip strength	28 (74)
	Pinch strength	12 (32)
	Stability	10 (26)
	Alignment	5 (13)
	Tripod grip strength	1 (3)
	Jebson-Taylor test	1 (3)
**Radiological**	**Any radiological outcome**	**34 (89)**
	Dislocation	20 (53)
	Loosening	17 (45)
	Migration/subsidence	12 (32)
	Fracture (at follow up)	10 (26)
	Radiographs (not otherwise specified)	9 (24)
	Heterotopic bone formation	5 (13)
	Implant fracture	5 (13)
	Erosions	3 (8)
	Osseointegration	3 (8)
	Joint survival	3 (8)
	Periprosthetic cysts	2 (5)
**Patient-reported**	**Any patient-reported outcome**	**34 (89)**
	Satisfaction	18 (47)
	Disabilities of the Arm, Shoulder and Hand (DASH) questionnaire	9 (24)
	Michigan Hand Outcomes Questionnaire	5 (13)
	“Function” not otherwise specified	4 (11)
	Patient Evaluation Measure (PEM)	4 (11)
	Canadian Occupational Performance Measure (COPM)	3 (8)
	Quick disabilities of the arm, shoulder and hand (quick DASH) questionnaire	3 (8)
	Appearance	3 (8)
	Patient-rated wrist evaluation (PRWE)	1 (3)
**Economic**	**Any economic outcome**	**1 (3)**
	Cost	1 (3)
**Adverse events**	**Any adverse event**	**35 (92)**
	Pain	32 (84)
	Revision	26 (68)
	Infection	25 (66)
	Deformity	19 (50)
	Reoperation (other than revision)	18 (47)
	Squeaking	14 (37)
	Intraoperative fracture	9 (24)
	Contracture/stiffness	9 (24)
	Wound healing problems	7 (18)
	Subluxation	6 (16)
	Adhesions	4 (11)
	Amputation	4 (11)
	Collateral ligament failure	3 (8)
	Cold intolerance	2 (5)
	Tendon rupture	1 (3)
	Triggering	1 (3)
	Bowstringing	1 (3)
	Finger ischaemia	1 (3)

### Clinical outcomes

The most commonly reported outcome was range of motion (ROM); in 37 (97%) of 38 included studies. Measurement of ROM used goniometry in 23 (61%) studies. Nine (24%) studies reported that the assessor of ROM was independent from the study authors. Grip and pinch strengths were reported in 28 (74%) and 12 (32%) studies respectively. Although ‘stability’ was reported in 10 studies, only four studies defined this outcome. All four of these studies used manual stress testing to assess stability. Three studies reported joints as either stable or unstable [26,33,38]. Storey et al reported that the number of degrees of coronal plane angulation was assessed under PIPJ stress, but did not clarify whether this process involved radiographs [45].

### Radiological outcomes

The use of radiographs was reported in 34 studies. Of these, nine (26%) did not specify any criteria for the interpretation of radiographs. Among 25/38 (66%) studies that reported specific radiological outcomes, criteria for the assessment of radiographs varied. For example, where ‘loosening’, ‘subsidence’ or ‘migration’ was reported, a range of assessment criteria were utilised. Herren et al [24] defined radiographic criteria which were used in further studies by Ono et al [34], Dickson et al [3] and Selig et al [55]. Sweets and Stern [20] reported a different system for the radiographic assessment of loosening, which was subsequently implemented by Watts et al [35] and further modified by Wagner et al [51].

### Patient-reported outcomes

There were nine patient-reported outcomes (PROs) across the 38 included studies, five of which were validated, upper-limb specific assessment tools [59,60]. The Disabilities of the Arm, Shoulder and Hand (DASH) questionnaire was used most frequently, in nine (24%) studies [25,31,33,40,42,43,46,55,57]. Of these, five reported DASH both at baseline and at a single time period post-operatively. Different follow-up time periods were used across these studies [25,31,33,43,46]. The Patient Evaluation Measure (PEM) was reported in four studies. The Michigan Hand Outcome Questionnaire (MHQ) was used by five studies [18,20,34,44,57]. Quick-DASH, a shortened questionnaire that has shown similar precision to DASH was used in three studies [61]. One study used the Patient-Rated Wrist Evaluation (PRWE) in addition to DASH [42]. The Canadian Occupational Performance Measure (COPM) was reported in three studies [31,43,46]. This outcome is not upper-limb specific–it evaluates changes in patient-specific occupational issues–but has been validated for use in osteoarthritis of the hand [62].

Patient satisfaction was reported in 18 (47%) studies. Of these, 16 (42%) reported how satisfaction was defined. Definitions and assessment methods varied across the studies. Methods included using a visual analogue scale (VAS) [3], Likert scale [25,40] or questionnaire [52] to rate how satisfied each patient was with the procedure and asking patients whether they would undergo the same procedure again [24]. Three studies reported “function”, without stating the use of a validated outcome tool [27,38,47].

### Process outcomes

No studies reported outcomes related to process, such as operating time or length of stay.

### Cost and economic outcomes

One paper reported the cost of pyrocarbon implants compared to silicone implants [41]. No analyses of cost effectiveness or other health economic evaluations were identified in the included studies.

### Adverse events

All included studies reported at least one adverse event. In total, 18 different adverse event outcomes were reported. Pain was most frequently reported, in 32 (84%) studies, with 26 (68%) using defined criteria. Twenty-one studies used a VAS to assess pain, of which four explicitly stated that patients completed the VAS themselves. Authors’ definitions of ‘early complications’ varied from three [37] to six [3] months post operatively. Twenty-eight studies reported surgical complications without stating a timescale. No study followed a standardised method for the classification or reporting of surgical complications.

### Implant removal

A total of 26 (68%) studies provided information on removal of implants. Reported reasons for implant removal and revision surgery included instability, loosening, stiffness and infection [35]. Two studies included a description of the device removal procedure [28,49].

## Discussion

This systematic review represents the first comprehensive analysis of how pPIPJa, an example of a surgical innovation, has been reported in the literature. Thirty-eight studies were identified, of which one was a RCT. Ethical issues were identified in many of the included studies, such as conflicting interests, failure to declare whether funding was received, and the unclear reporting of patient consent. Six studies reported that the intervention was modified during the study period, however it was unclear whether patients were informed of this change in practice. Reporting of operator and centre expertise was limited. Reporting of surgical technique and postoperative rehabilitation varied widely among included studies, rendering intervention replication and comparison with other published data difficult. Outcome selection and definition were inconsistent, limiting scope for evidence synthesis. No study used a standardised system for classifying surgical complications and the majority lacked a reporting timescale. Overall, this review identified widespread issues with the reporting of pPIPJa. It adds to the body of evidence supporting the urgent need for improvements in transparency in the development and reporting of novel invasive devices.

This study supplements four systematic reviews that focused on pPIPJa outcomes [63–66]. In 2008, Squitieri and Chung compared outcomes and complications between vascularised toe-joint transfer, silicone arthroplasty and pPIPJa for posttraumatic finger joint reconstruction [56]. The authors concluded that pPIPJa may be associated with higher rates of major complications, although only two pPIPJa papers were included. Chan et al (2013) compared pPIPJa to silicone PIPJ arthroplasty [64]. Their review included a further four comparative studies but no RCTs, and concluded that insufficient data was available. The report raised concerns about high complication rates with pPIPJa; revision and salvage procedure rates were reported to be nearly four times higher with pPIPJa than silicone arthroplasty [64]. More recent reviews have aimed to stratify complication and revision rates according to implant type, and to compare ulnar digits with the ring finger [65,66]. Our study differed from existing reviews in its focus on reporting, rather than on outcomes. We identified that the conclusions of included studies were frequently conflicting. Eight papers recommended the adoption of pPIPJa into routine practice while six advised against further use of the implant [20,25,39,41,42,44]. In 2013, Chan et al recommended that studies of PIPJ arthroplasty should use validated quality-of-life scales and economic evaluation. However, only Van Nuffel et al (2014) have since reported implant cost; no included studies used quality of life outcomes [41]. Despite these important issues, pPIPJa implants continue to be marketed for use in routine practice [67]. This is concerning in the context of wider issues around the governance of medical devices. Metal-on-metal hip implants, for example, have been associated with local tissue damage and systemic reactions, while transvaginal mesh has caused serious health problems, despite existing regulatory processes being followed [68].

Currently, data from pre-marketing studies is not publicly available for scrutiny. This means that implants may enter the marketplace and be widely used with limited knowledge about long term outcomes. Historically, Food and Drug Administration (FDA) approval for ‘medium-risk devices’, which included orthopaedic implants, was based on pre-clinical studies only [69]. To improve regulation of medical devices without stifling innovation and compromising patients’ access to treatments, a graduated model of approval has been recommended [70]. The IDEAL-D (Idea, Development, Exploration, Assessment, Long term study-Devices) framework comprises stepwise guidance for the scientific evaluation of devices. It directs innovators and academics to conduct research that is transparent, robust, and appropriate to a maturing intervention’s stage of development. A central principle of IDEAL-D is the importance of developing co-operative international registries to prospectively monitor all devices, thereby expediting the detection of harm [70]. There has been a call for implants to be tested in RCTs prior to being made routinely available, in line with IDEAL-D recommendations and existing pharmaceutical governance [70,71]. In the USA, invasive devices are now subject to a pivotal clinical study as part of the FDA’s premarket approval pathway. The UK Medicines and Healthcare products Regulatory Agency (MHRA) does not specify the type of study required to achieve device approval, but the majority do not include a control group [72]. Pressure for reform is mounting. The Royal College of Surgeons of England (2018) has urged the government to address the “lax regulation system governing medical devices, including a compulsory registry of all new implants” [71].

Currently, it is mandatory to register the implantation, or subsequent removal of hip, knee, ankle, shoulder and elbow replacements with the UK National Joint Registry (NJR) [73]. The UK Hand Registry has included joint replacement procedures since 2017, but registration of procedures by surgeons is voluntary. A major inquiry into medicines and device regulation in the United Kingdom concluded that a register of all medical devices should be established, and inclusion on the register would be a condition of selling the device in the UK [74]. Compulsory registration is crucial to ensure transparency, accountability and device equivalence. In the high-profile case of metal-on-metal hip implants, for example, NJR data was instrumental; firstly by triggering several MHRA Medical Device Alerts from 2008 onwards, secondly in reporting early failure of Articular Surface Replacement–a specific device–in 2010, and subsequently in demonstrating that, rather than being implant specific, large-head metal-on-metal failure is a class effect [70,75,76]. Compulsory registration may not prevent device failure, but does help to identify patient harm at an early stage and ensure that practice changes. A major challenge with registries, however, is collecting accurate prospective data.

Our study tracked the introduction and evolution of pPIPJa from first published description to present day. Despite this, there were limitations. Exclusion of non-English language papers may mean that additional findings were missed. Notably, a 2005 study published in German was excluded [77]. Data were extracted from the papers verbatim, and because this review focused on reporting, authors were not contacted for further information. Although all included studies were conducted in high-income countries, the model of health service provision (e.g. private vs. public sector) was not formally assessed in this review. Twenty-two of the 38 included studies were from USA, where the implant is manufactured and private healthcare is common. It is possible that systematic differences between the characteristics of patients and methods of care delivery in private and public sectors may have introduced important factors, including bias, that were not analysed in this study.

Overall, this systematic review demonstrates that the conclusions of included studies were not based on the evidence within the papers, which had methodological weaknesses. Our results are consistent with existing evidence that surgical innovations have been poorly reported [72]. Future work is needed to change how devices are used and evaluated after approvals, to allow transparent incremental evidence to be created to inform clinical practice and decision-making.

## Supporting information

S1 Checklist PRISMA checklist. (DOC)

S1 File Search strategy. (PDF)
