# Supplementary material for: Systematic review of the introduction, early phase study and evaluation of pyrocarbon proximal interphalangeal joint arthroplasty
Source: PLoS One. 2021 Oct 19;16(10):e0257497. doi: 10.1371/journal.pone.0257497 (PMC8525747; doi:10.1371/journal.pone.0257497)
Supplement: S1 File — (PDF) [file pone.0257497.s002.pdf]

S1. Search strategy

Searches will be undertaken in OVID SP versions of Medline, and EMBASE, SCOPUS, Web of Science, BIOSIS, CINAHL, the Cochrane Central Register of Controlled Trials. Only articles published in English language will be included. Conference abstracts will not be included

Database: Ovid MEDLINE(R) and Epub Ahead of Print, In-Process & Other Non-Indexed Citations and Daily <1946 to November 01, 2020>
Search Strategy:
----
1 finger joint/ or palmar plate/
2 PIPJ.ti,ab,kf.
3 PIP.ti,ab,kf.
4 finger joint*.ti,ab,kf.
5 (proximal interphalangeal or proximal inter-phalangeal).ti,ab,kf.
6 (finger* adj3 joint*).ti,ab,kf.
7 1 or 2 or 3 or 4 or 5 or 6
8 arthroplasty/ or replacement, finger/ or hemiarthroplasty/
9 (arthroplasty or replacement).ti,ab,kf.
10 (prosthes* or implant*).ti,ab,kf.
11 8 or 9 or 10
12 7 and 11
13 limit 12 to english language

****
